# The Changing Role of Sound‐Symbolism for Small Versus Large Vocabularies

**DOI:** 10.1111/cogs.12565

**Published:** 2017-12-12

**Authors:** James Brand, Padraic Monaghan, Peter Walker

**Affiliations:** ^1^ Department of Psychology Lancaster University; ^2^ Department of Psychology of Language Max Planck Institute for Psycholinguistics; ^3^ Department of Psychology Sunway University

**Keywords:** Sound‐symbolism, Language learning, Vocabulary development, Language evolution

## Abstract

Natural language contains many examples of sound‐symbolism, where the form of the word carries information about its meaning. Such systematicity is more prevalent in the words children acquire first, but arbitrariness dominates during later vocabulary development. Furthermore, systematicity appears to promote learning category distinctions, which may become more important as the vocabulary grows. In this study, we tested the relative costs and benefits of sound‐symbolism for word learning as vocabulary size varies. Participants learned form‐meaning mappings for words which were either congruent or incongruent with regard to sound‐symbolic relations. For the smaller vocabulary, sound‐symbolism facilitated learning individual words, whereas for larger vocabularies sound‐symbolism supported learning category distinctions. The changing properties of form‐meaning mappings according to vocabulary size may reflect the different ways in which language is learned at different stages of development.

## 1 Introduction

The vocabulary that an adult acquires largely comprises arbitrary words (De Saussure, 1916; Hockett, 1960). However, recent interest in the presence of non‐arbitrary form‐meaning mappings has challenged the traditional view that arbitrariness should be considered a design feature of language (Dingemanse, Blasi, Lupyan, Christiansen, & Monaghan, 2015). Perhaps, the most well‐documented example of a sound‐symbolic relation between form and meaning is the “bouba‐kiki” effect (Köhler, 1929, 1947; Ramachandran & Hubbard, 2001), where a specific preference is observed for matching particular sounds in non‐words with either rounded (“bouba”) or spiky (“kiki”) shapes (Bremner et al., 2013; Cuskley, Simner, & Kirby, 2015; Dingemanse, Schuerman, Reinisch, Tufvesson, & Mitterer, 2016; Kovic, Plunkett, & Westermann, 2010; Maurer, Pathman, & Mondloch, 2006; Ozturk, Krehm, & Vouloumanos, 2013; Walker et al., 2010).

Sound‐symbolism may be particularly useful for assisting in learning word‐referent mappings at an early stage of language development. Given that a learner is confronted by the difficult task of determining form‐meaning mappings (Harnad, 1990; Quine, 1960), sound‐symbolism may assist children to learn that words have reference because of an inherited understanding of cross‐sensory correspondences (Imai & Kita, 2014; Imai, Kita, Nagumo, & Okada, 2008; Kantartzis, Imai, & Kita, 2011; Maurer et al., 2006; Nygaard, Cook, & Namy, 2009; Walker et al., 2010). Thus, learners are provided with information about the meaning of the word by incorporating signification within the actual form used, enabling the learner to realize that the form is potentially referential and, further, what the referent actually is (Ramachandran & Hubbard, 2001; Spector & Maurer, 2009).

The importance of sound‐symbolism for early language development is supported by studies of systematicity, a form of non‐arbitrariness that describes the link between sound patterns in the language and shared meanings through statistical relationships (see Dingemanse et al., 2015). In an analysis of the vocabulary of English, non‐arbitrariness was found to be more prevalent for the words children acquire earlier in language (Monaghan, Shillcock, Christiansen, & Kirby, 2014). For the words children learn between the ages of 2 and 5, there is greater systematicity between form and meaning of words than expected by chance. Similarly, Perry, Perlman, and Lupyan (2015) found that words rated as iconic by adult participants, that is, rated highly as “words that sound like what they mean,” were more likely to be those that children acquire earlier in vocabulary development.

However, the sound‐symbolism present in the early vocabulary diminishes in the later vocabulary: In Monaghan et al.'s (2014) analysis, from ages 7 onward, there tends to be greater arbitrariness than expected by chance in form‐meaning mappings. Thus, to understand the role of sound‐symbolism in language development, it is necessary to understand when sound‐symbolism is advantageous for the learner, and when it is not.

Gasser (2004) predicted that arbitrariness in sound‐meaning mappings should be increasingly beneficial for learning as the vocabulary size increases. If word forms contain sound‐symbolism, then this restricts the possibilities for new words to be interleaved with the representations of previously acquired words, whereas arbitrary relations enable greater flexibility in forming the new word's mapping. Monaghan, Christiansen, and Fitneva (2011) also predicted from computational modeling that arbitrary relations ought to be advantageous for learning larger vocabularies because they reduce the likelihood of ambiguity being introduced into the expression, whereby similar‐sounding word forms are used to represent different meanings, for example, dog and cog. Thus, sound‐symbolism limits the distinctiveness between words with similar meanings, which is not problematic when there are just a few words in the vocabulary, but which becomes an increasing strain on form‐meaning mapping formation as the sound space becomes populated with a larger vocabulary. However, these benefits of arbitrariness for learning larger vocabularies over smaller vocabularies are yet to be tested experimentally. Thus, we predict that sound‐symbolism is beneficial for learning individual sound to meaning mappings for a small vocabulary, but that this facilitation will reduce with a larger vocabulary.

Although there is increasing arbitrariness at the individual word level for the growing vocabulary (Monaghan et al., 2014; Perry et al., 2015), systematicity at the *category* level is observable across the whole vocabulary. Kelly (1992) showed that there is a systematic correspondence between the sounds of words and their grammatical category which applies cross‐linguistically (Monaghan, Christiansen, & Chater, 2007). The same idea that phonology can be used advantageously to provide category‐level information had driven historic efforts to create entirely systematic, universal languages, whereby meaning could be comprehended simply from the form being expressed (e.g., Wilkins, 1668).

Monaghan, Mattock, and Walker (2012) tested whether learning could be supported by systematicity at the category level. They trained participants to map between 16 non‐words and meanings drawn from two shape categories. They varied the extent to which there was a systematic or arbitrary relation between the sounds of the words and the category distinction. They found that systematicity facilitated learning of the broader category distinctions between words (see also Farmer, Christiansen, & Monaghan, 2006). Thus, although sound‐symbolism may be useful for individual word learning for small vocabularies, sound‐symbolism ought to be beneficial for learning category distinctions for larger vocabularies.

**Figure 1:**
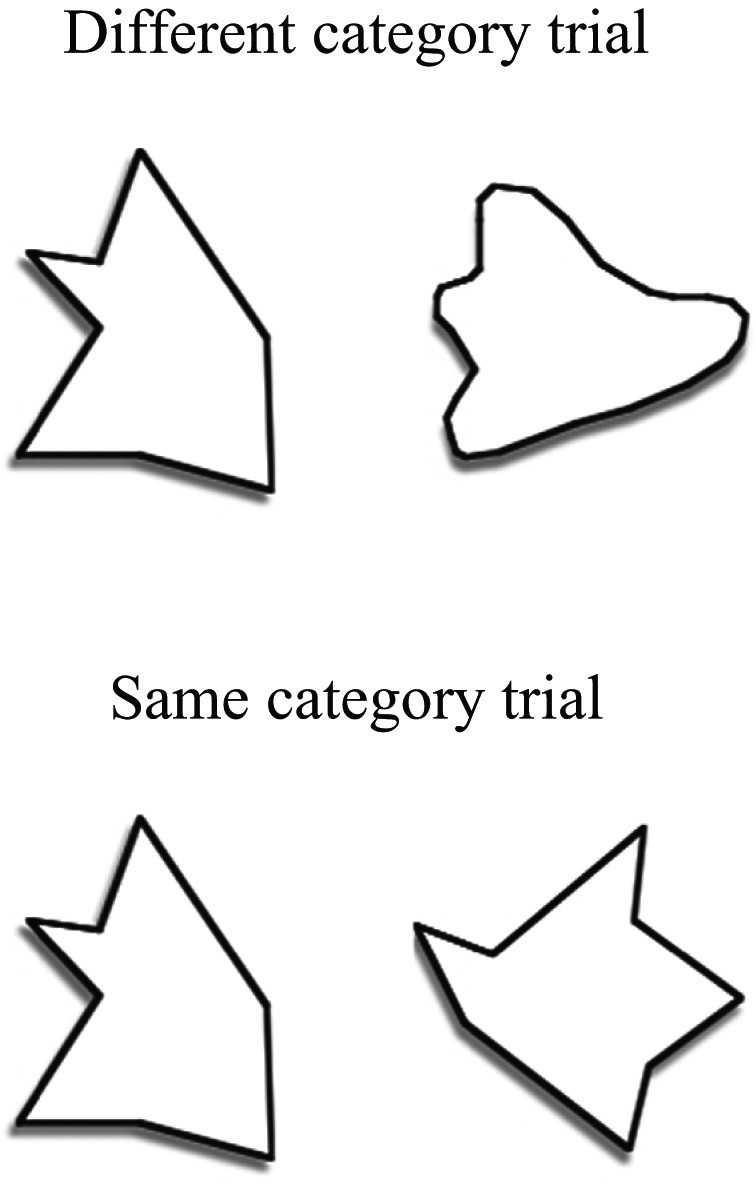
Examples of a same and different category trial. A congruent mapping would pair a plosive word, for example, /bIk/, to the angular shape, while an incongruent mapping would pair a plosive word with the rounded shape.

In the experiment reported here, we tested the effect of sound‐symbolism on learning individual word meanings and category distinctions for different sizes of vocabulary. Adult participants were trained to learn word‐referent mappings, where referents were either rounded or angular visual shapes. Mappings were either congruent with sound‐symbolism, where the word was paired with an object to reflect previously established sound‐symbolic relations, or incongruent, where the mapping was inconsistent with these relations. Learning trials varied in terms of whether the participant had to discriminate between choices from the two different shape categories (e.g., one angular and one rounded shape were presented), or whether the choices were from the same shape category ensuring that category‐level information was not available to support the decision (e.g., both angular) (see Fig. 1).

## 2 Method

### 2.1 Participants

Seventy‐two undergraduate students from Lancaster University, with a mean age of 18.7 years (*SD* = 0.8, range 17–21) participated. All participants spoke proficient English (55 had English as a first language). Informed consent was collected from each participant, and ethical approval was obtained from Lancaster University's ethics committee.

### 2.2 Materials

For the visual stimuli, 16 different shapes were constructed which were either rounded or angular in shape (eight shapes for each category). Shapes were similar in terms of perceived size and complexity in terms of numbers of protuberances (see Monaghan et al., 2012, for details of the controls).

For the auditory stimuli, 16 different monosyllabic consonant‐vowel‐consonant non‐words were recorded by a native English speaker in a monotone. For eight of the non‐words, plosives were used for the consonants (/k/,/g/,/t/,/d/,/p/,/b/) in both onset and coda positions. Continuants consisting of nasals, liquids, and approximants (/m/,/n/,/ŋ/,/l/,/ɹ/,/w/), comprised the onsets and codas for the remaining eight non‐words. Each non‐word contained a vowel chosen from one of the following four sounds (/æ/,/ɛ/,/ɪ/,/ɒ/). Each vowel was used an equal number of times within the sets of rounded and angular non‐words. The full list of non‐words used can be found in Table 1.

**Table 1:** List of phonetically transcribed words used during the experiment

Continuant Words	Plosive Words
/mɒŋ/ /nɪm/ /læn/ /ɹɛŋ/ /wɒl/ /wɛm/ /ɹɪn/ /næl/	/kɪb/ /gæt/ /tɛg/ /dɒp/ /pɛd/ /bɪk/ /tɒb/ /kæg/

**Figure 2:**
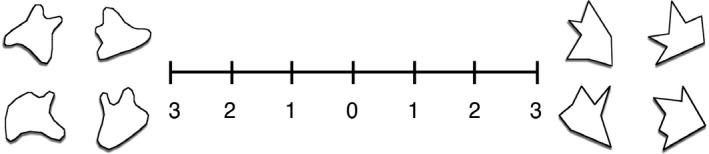
Example of Likert scale item for correspondence between word and rounded or angular shapes. Rounded shapes were presented on the left side of the scale for half the trials and on the right for the other half.

To ensure that the sounds used were reliably sound‐symbolic, 22 additional participants completed a short questionnaire rating the strength with which they felt each sound corresponded to rounded or spiky shapes, which were illustrated on either side of a 7‐point scale. The mid‐point of the scale consisted of “0” for no correspondence, and then ran from “1” for weak, “2” for medium, and “3” for strong correspondence in each direction (an example item is shown in Fig. 2). Ratings indicating an angular shape preference were coded as negative values. Plosive non‐words were judged to correspond more closely to angular than rounded shapes (mean rating = −0.58, *SD* = 1.49), whereas continuant non‐words more closely corresponded to rounded shapes (mean rating = 0.18, *SD* = 1.37), and these scores were significantly different, *t*(672.55) = −6.867, *p *<* *.001.

For the vocabulary learning task, sounds were mapped to the shapes in two different ways for each participant. Half the mappings were congruent with previous sound‐symbolic studies of phoneme to shape mappings (Fort, Martin, & Peperkamp, 2015; Nielsen & Rendall, 2012), where rounded shapes were mapped to the continuant non‐words, while angular shapes were mapped to the plosive non‐words. The other half of the mappings were incongruent, which paired rounded shapes with plosives and angular shapes with continuants. Participants were exposed to an equal number of congruent and incongruent trials during the experiment.

The small vocabulary condition presented four rounded and four angular images and four plosive and four continuant non‐words, selected randomly from the set of 16 images and 16 non‐words for each participant. The medium size vocabulary condition selected 12 images and 12 non‐words from the set of 16. The large vocabulary size utilized all 16 images and non‐words, and was thus similar in design to Monaghan et al. (2012).

### 2.3 Procedure

A cross‐situational learning paradigm was used in the experiment (see Smith & Yu, 2008). Participants heard a sound and viewed two shapes side by side on a computer screen, and were required to decide which shape they thought the sound referred to, pressing “1” or “2” on a computer keyboard to select the left or right shape, respectively. One image had been pre‐selected to be the target, which always co‐occurred with the spoken word, and one was the foil, which was one of the other images in the set to be learned. Positions of targets and foils were counterbalanced within blocks of trials, and no feedback was given.

The foil was a shape that was either from the same shape category as the target, or from the different shape category, allowing a test of whether a broad categorical distinction was being learned, or the meanings of individual words (see Fig. 1 for an example). Learning is therefore tested by ability to discriminate between two alternatives, which is a standard method for testing word learning (e.g., Horst, Samuelson, Kucker, & McMurray, 2011). There were four blocks of training, within which each mapping was presented four times. As the number of mappings varied in each vocabulary condition, the number of trials per block also varied: 32 trials per block for the small, 48 trials for the medium, and 64 trials for the large vocabulary condition.

## 3 Results

In the analysis conducted on the data,^1^ we modeled the probability (log odds) of response accuracy, accounting for the variation across participants and stimuli. Observations were clustered for each participant and stimulus; therefore, we performed a series of generalized linear mixed‐effects models (Baayen, 2008; Jaeger, 2008), specifying first the random effects of subject and individual stimulus (i.e., word sound). Then, we considered the effect of experimental condition (vocabulary size), the effect of block over the course of the experiment, the effect of learning trial type (same or different category presentation), and also the effect of congruency. We then considered the interaction between vocabulary size, same versus different shape condition, and congruency. After adding each fixed effect to the model, we ran likelihood ratio test comparisons, comparing the new model to the previous one. This showed whether the inclusion of the new term significantly improved the fit of the model.

**Figure 3:**
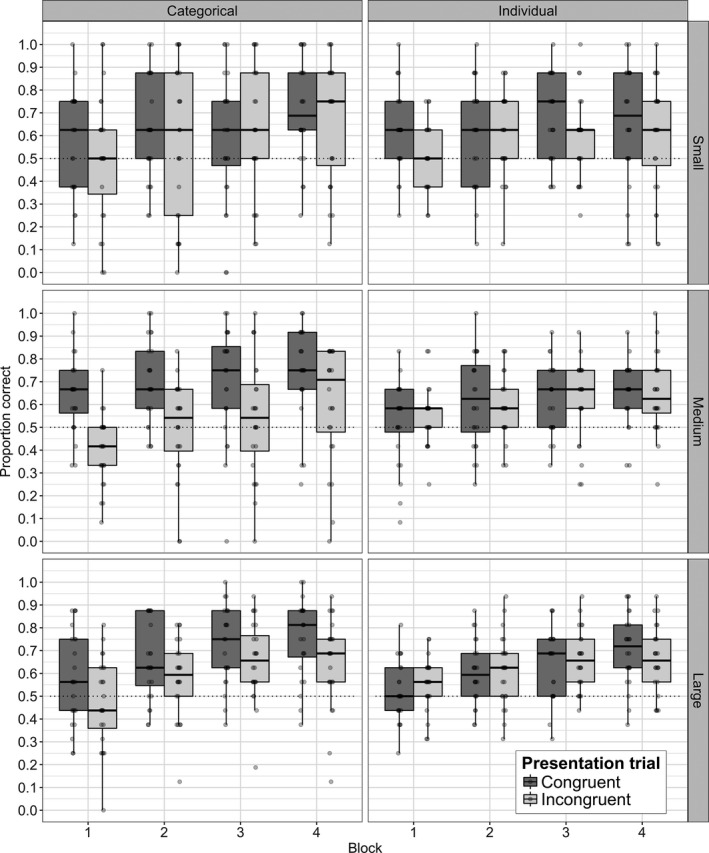
Proportion of correct responses by block, for same and different category presentations, by vocabulary size condition. Dots represent individual subject data. Dotted line shows 50% chance level.

Adding the effect of vocabulary size to a model with just random effects did not significantly improve the fit of the model, χ^2^(2) = 0.97, *p *=* *.62. The inclusion of the effect of block significantly improved the fit of the model, χ^2^(3) = 153.1, *p *<* *.001, and this effect was found to be positive, indicating that performance over the course of the experiment improved: estimated intercept log odds for the model = 0.20, *SE* = 0.02, *z *=* *12.33, *p *<* *.001, see Fig. 3. Additionally, including the interaction term of vocabulary size X congruency X categorical/individual learning also significantly improved model fit, χ^2^(8) = 31.5, *p *<* *.001. This indicated that the effect of sound‐symbolism for the categorical and individual learning tasks varied as a function of vocabulary size. The interaction was significant in a positive linear fit (estimate = 0.39, *SE* = 0.13, *z *=* *2.98, *p *=* *.003). Full details of the model selection can be found in Table 2 and the final model summary in Table 3.

**Table 2:** Main model selection

Model	Fixed Effects	AIC	BIC	LogLik	χ^2^	*p*	Preferred Model
1	−	18,201	18,223	−9,097.4	−	−	−
2	1 + condition	18,204	18,241	−9,096.9	0.9655	0.6171	1
3	1 + block	18,051	18,081	−9,021.3	152.14	<0.0001	3
4	3 + congruency	17,995	18,033	−8,992.5	57.633	<0.0001	4
5	4 + same or different shape condition	17,996	18,042	−8,992.2	0.4949	0.4817	4
6	4 + condition × congruency	17,994	18,062	−8,988.1	8.7971	0.0664	4
7	4 + condition × same or different shape condition	18,002	18,078	−8,991.2	2.5736	0.7654	4
8	4 + congruency × same or different shape condition	17,962	18,015	−8,974.1	36.753	<0.0001	8
9	8 + condition × congruency × same or different shape condition	17,947	18,060	−8,958.3	31.511	<0.001	9

The table provides Bayesian Information Criterion (BIC), Akaike Information Criterion (AIC), and log‐likelihood (logLik) for several potential models fit to the data for Experiment 1. For all models, the glmer() call was Response [Fixed effects]+(1|Subject)+(1|Sound) and fit a binomial model (i.e., all models used the same outcome variable and random effects).

**Table 3:** Summary of the generalized linear mixed‐effects model of (log odds) accuracy of response over blocks, experimental conditions, congruency, and same or different shape condition

Fixed Effects	Estimated Coefficient	*SE*	Wald Confidence Intervals 2.50% 97.50%		*z*	*P*r(>|*z*|)
(Intercept)	0.2388	0.0720	0.0978	0.3798	3.3180	0.0009
Block effect	0.1983	0.0161	0.1667	0.2298	12.3280	<0.0001
Congruency (congruent vs. incongruent)	−0.4736	0.0544	−0.5802	−0.3671	−8.7120	<0.0001
Same or different shape condition (categorical vs. individual)	−0.2088	0.0536	−0.3139	−0.1038	−3.8980	<0.0001
Experimental condition (linear)	−0.1619	0.0973	−0.3526	0.0289	−1.6630	0.0963
Experimental condition (quadratic)	−0.1521	0.0964	−0.3410	0.0368	−1.5780	0.1145
Congruency:same or different shape condition interaction	0.3694	0.0746	0.2232	0.5156	4.9530	<0.0001
Experimental condition (linear):congruency interaction	0.1672	0.0936	−0.0162	0.3506	1.7870	0.0740
Experimental condition (quadratic):congruency interaction	0.4260	0.0902	0.2492	0.6027	4.7230	<0.0001
Experimental condition (linear):same or different shape condition interaction	0.2543	0.0942	0.0696	0.4390	2.6990	0.0070
Experimental condition (quadratic):same or different shape condition interaction	0.2384	0.0912	0.0597	0.4170	2.6150	0.0089
Experimental condition (linear):congruency:same or different shape condition interaction	−0.3918	0.1316	−0.6497	−0.1340	−2.9780	0.0029
Experimental condition (quadratic):congruency:same or different shape condition interaction	−0.5171	0.1266	−0.7652	−0.2689	−4.0840	<0.0001
Random effects						
Groups	Name	Variance	Std.Dev.			
Subject effect on intercepts	(Intercept)	0.12	0.35			
Item effect (objects) on intercepts	(Intercept)	0.01	0.09			
	AIC	BIC	logLik	deviance		
	17,946.7	18,059.7	−8,958.3	17,916.7		
						

Note

There were 13,824 observations, 72 participants, and 16 sound stimuli. R syntax for final model: glmer(accuracy ~ block + condition + congruency + learning_type + condition*congruency*learning_type + (1 | Subject) + (1|Sound).

**Figure 4:**
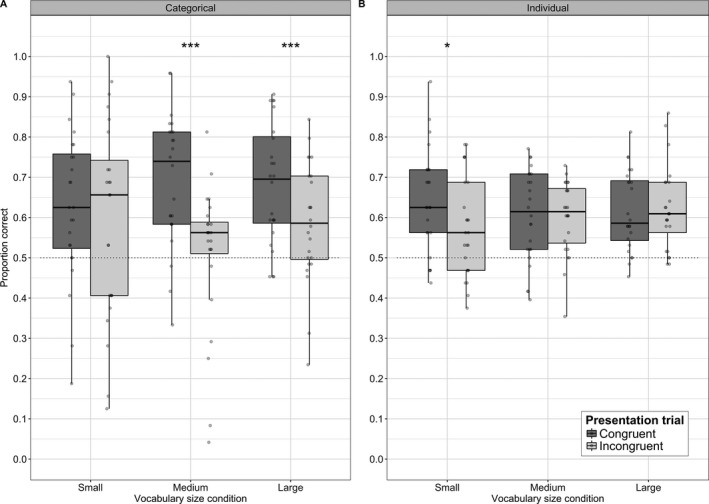
Proportion of correct responses in (A) different category presentation trials (categorical learning) and (B) same category presentation trials (individual word learning). Dots represent individual subject data. *p < .05 and ***p < .001.

To understand this three‐way interaction, we tested models investigating performance for categorical and individual word‐learning trials separately, allowing us to explore the two‐way interactions between vocabulary size and congruency. For categorical trials, the inclusion of the interaction term as both a linear and quadratic effect significantly improved model fit, χ^2^(4) = 24.2, *p *<* *.001. In follow‐up one‐way analyses, congruency improved model fit for the medium and large vocabulary sizes, χ^2^(1) = 86.399, and χ^2^(1) = 30.437, both *p *<* *.001. However, for the small vocabulary size, congruency did not significantly improve model fit, χ^2^(1) = 2.3061, *p *=* *.13, see Fig. 4. Thus, sound‐symbolism boosted categorization only for the medium and large vocabularies. With more items within the category for the medium and large vocabularies, than within the small vocabulary, the effect of category‐level sound symbolism in these larger vocabularies appears to have been strengthened.

For individual word‐learning trials, the linear and quadratic interaction terms did not improve model fit, χ^2^(5) = 7.5, *p *=* *.19, although the linear interaction effect was significant in the model, *p *=* *.017. In follow‐up one‐way analyses, congruency improved model fit for the small vocabulary size, χ^2^(1) = 6.5879, *p *=* *.01, whereas for the medium and large vocabulary sizes, congruency did not significantly improve model fit, χ^2^(1) = .012, *p *=* *.91 and χ^2^(1) = .0561, *p *=* *.81, respectively, see Fig. 4. Thus, sound‐symbolism promoted learning individual word‐shape mappings, but only for the small vocabulary.

## 4 Discussion

This study demonstrated one of the reasons why sound‐symbolism is evident in early vocabulary development but why arbitrariness is dominant for later vocabulary development (Massaro & Perlman, 2017; Monaghan et al., 2014; Perry et al., 2015). We showed that when the vocabulary is small, as in the first stages of vocabulary acquisition, sound‐symbolism is advantageous for learning the meanings of individual words. Thus, sound‐symbolism can effectively be incorporated into the vocabulary structure to support acquisition of word‐referent mappings (Imai et al., 2008; Kantartzis et al., 2011; Nygaard et al., 2009). However, for the larger vocabulary sizes, the advantage at the individual word level for sound‐symbolism was not observed, instead sound‐symbolism was advantageous only for learning category distinctions. This provides a potential explanation for why vocabulary acquired later in life tends not to contain sound‐symbolism for individual words (Monaghan et al., 2014) but does demonstrate systematicity between sounds and categories of words (Farmer et al., 2006; Kelly, 1992; Monaghan et al., 2007).

These findings highlight the potential benefits of sound‐symbolism for learning at different stages of vocabulary development. When a language learner is initially acquiring a vocabulary, sound‐symbolism may provide an effective, even essential, scaffold that aids the acquisition of the first words in the vocabulary (Kantartzis et al., 2011). This could then provide a bootstrapping effect, allowing for a more densely populated vocabulary to be acquired subsequently (Imai & Kita, 2014). For a larger vocabulary, an arbitrary system becomes more suited for the demands of communication, with non‐arbitrariness applying only at the level of distinguishing categories rather than individual meanings. Thus, the general processing constraints introduced by a growing vocabulary are reflected in children's vocabulary acquisition. Language appears to be structured to promote sound‐symbolic mappings early on in vocabulary learning, but, as the vocabulary expands, arbitrary mappings become dominant as the communicative system demands greater expressivity and signal efficiency.

Our demonstration of the changing effects of sound‐symbolism as vocabulary size increases provides the first behavioral demonstration of predictions derived from theoretical and computational modeling, highlighting the advantages of arbitrariness for larger vocabularies and sound‐symbolism for when the vocabulary is smaller. Our work thus provides an answer not only to the question as to why sound‐symbolism is prevalent in early vocabulary, but also why arbitrariness is dominant as the vocabulary size increases. We see these questions as related and have provided a single framework, grounded in computational theories of cross‐modal mappings (e.g., Gasser, 2004), that identifies the vital role of both systematic and arbitrary mappings in the vocabulary of a language. We have shown that observations of sound‐symbolism being more prominent in early‐ than late‐acquired vocabulary in natural language studies are supported by the learning advantages observed with different vocabulary sizes. This is also consistent with views of the evolution of language, whereby a sound‐symbolic system might have been key during a proto‐language stage (e.g., Ramachandran & Hubbard, 2001), but as language evolved under communicative pressures of increasing expressivity, arbitrariness came to dominate the communicative system.

## Note

1 Data available at https://git.io/v5BXJ
